# Saudi Community-Based Screening Study on Genetic Variants in *β*-Cell Dysfunction and Its Role in Women with Gestational Diabetes Mellitus

**DOI:** 10.3390/genes14040924

**Published:** 2023-04-16

**Authors:** Amal F. Alshammary, Malak Mohammed Al-Hakeem, Imran Ali Khan

**Affiliations:** 1 Department of Clinical Laboratory Sciences, College of Applied Medical Sciences, King Saud University, Riyadh 11433, Saudi Arabia; 2 Department of Obstetrics and Gynecology, College of Medicine, King Khalid University Hospital, Riyadh 11451, Saudi Arabia

**Keywords:** GDM, rs7903146-TCF7L2, rs2237892-KCNQ1, rs5219-KCNJ11, Saudi women

## Abstract

Background: Diabetes (hyperglycemia) is defined as a multifactorial metabolic disorder in which insulin resistance and defects in pancreatic β-cell dysfunction are two major pathophysiologic abnormalities that underpin towards gestational diabetes mellitus (GDM). *TCF7L2*, *KCNQ1*, and *KCNJ11* genes are connected to the mechanism of β-cell dysfunction. The purpose of this study was to investigate the genes associated with β-cell dysfunction and their genetic roles in the rs7903146, rs2237892, and rs5219 variants in Saudi women diagnosed with type 2 diabetes mellitus and GDM. Materials and Methods: In this case-control study, 100 women with GDM and 100 healthy volunteers (non-GDM) were recruited. Genotyping was performed using polymerase chain reaction (PCR), followed by restriction fragment length analysis. Validation was performed using Sanger sequencing. Statistical analyses were performed using multiple software packages. Results: Clinical studies showed a β-cell dysfunction positive association in women with GDM when compared to non-GDM women (*p* < 0.05). Both rs7903146 (CT vs. CC: OR-2.12 [95%CI: 1.13–3.96]; *p* = 0.01 & T vs. C: (OR-2.03 [95%CI: 1.32–3.11]; *p* = 0.001) and rs5219 SNPs (AG vs. AA: OR-3.37 [95%CI: 1.63–6.95]; *p* = 0.0006 & G vs. A: OR-3.03 [95%CI: 1.66–5.52]; *p* = 0.0001) showed a positive association with genotype and allele frequencies in women with GDM. ANOVA analysis confirmed that weight (*p* = 0.02), BMI (*p* = 0.01), and PPBG (*p* = 0.003) were associated with rs7903146 and BMI (*p* = 0.03) was associated with rs2237892 SNPs. Conclusions: This study confirms that the SNPs rs7903146 (*TCF7L2*) and rs5219 (*KCNJ11*) are strongly associated with GDM in the Saudi population. Future studies should address the limitations of this study.

## 1. Introduction

Diabetes mellitus has become a global health problem. Chronically abnormal blood sugar levels are a hallmark of this potentially lethal metabolic condition [1]. According to the International Diabetes Federation, approximately 700 million adults aged 18–99 will be diagnosed with diabetes worldwide by 2045 [2]. Type 2 diabetes mellitus (T2DM) is a common form of diabetes, accounting for 90–95% of cases, with the remainder classified as T1DM, gestational diabetes mellitus (GDM), and other types of diabetes [3]. Obesity is a risk factor and one of the common factors for developing both T2DM and GDM because obesity alters adipokine secretion, leading to insulin resistance, which is a definite association between obesity and diabetes [4]. Changes in lifestyle and diet are major contributors to the T2DM/GDM pandemic, but inheritable factors account for a significant portion of phenotypic variance [5]. The underlying pathophysiologies of GDM and T2DM are increased insulin resistance and defects in insulin secretion [6]. GDM diagnosed when glucose intolerance is initially present during pregnancy is classified as a perinatal pathology, with a global prevalence of 5–20% in pregnant women. It is triggered by higher weight gain, body mass index (BMI), advanced maternal age, conceiving age, and metabolic disorders [7]. Recurrent GDM during subsequent pregnancies is most strongly associated with a strong family history of T2DM and GDM [8].

Until now, genome-wide association studies (GWAS) have identified approximately 120 susceptibility loci in T2DM; whereas functional defects have yet to be assigned, many of these loci point to primary defects in β-cell function rather than insulin resistance [9]. In terms of disease pathophysiology, a breakdown in the feedback loops between insulin action and secretion results in abnormally high blood glucose levels. β-cell failure causes a decrease in insulin secretion, which is essential for maintaining consistent blood sugar levels, although insulin resistance also plays a role, leading to increased glucose synthesis in the liver and diminished glucose uptake by adipose tissue [10]. The gradual loss of β-cell mass and function is an early and observable aspect of the natural pathway of diabetes [11]. Failure of pancreatic β-cells can lead to the interaction of genetic and acquired factors that are important in the onset and progression of T2DM [12]. Human genetics is an invaluable resource for the study of β-cell dysfunction. Various Mendelian diabetes studies have resulted in significant advances in our understanding of β-cell biology and delivery of approved therapeutic targets. Although the initial T2DM susceptibility variants took longer to translate into clinical practice, documented studies have made significant progress in identifying the processes underlying several common and low-frequency susceptibility variants [13]. Glucose sensing, sensitivity to secretory potentiators and inhibitors, proinsulin synthesis and processing, and insulin granule exocytosis are examples of mechanisms that can be dysregulated in β-cells [14].

Only a few genes contribute to the study of maternal metabolism during pregnancy. Transcription factor 7 like 2 (*TCF7L2*), potassium inwardly rectifying channel Subfamily J member 11 (*KCNJ11*), and potassium Voltage-Gated Channel Subfamily Q Member 1 (*KCNQ1*) encoding genes are commonly associated with β-cell dysfunction, and a combination of insulin resistance and β-cell dysfunction can frequently result in persistent hyperglycemia [15]. Single nucleotide polymorphisms (SNPs) occur in the DNA sequences of people at individual nucleotide(s) base pairs.

In Saudi Arabia, the prevalence of diabetes was found to be 24%, and the prevalence of GDM was found to be 24.2% [16]. Research on women with GDM in Saudi Arabia has been stagnating, especially in the realm of molecular studies. Notably, the discovery of rs7903146, rs2237892, and rs5219 SNPs began more than a decade ago, and no studies have been conducted in Saudi women diagnosed with GDM. Therefore, in this study, we investigated the molecular role of rs7903146 (*TCF7L2*), rs2237892 (*KCNQ1*), and rs5219 (*KCNJ11*) SNPs in Saudi women diagnosed with GDM.

## 2. Materials and Methods

### 2.1. Sanction of Ethical and Consent Approvals

The Institutional Review Board of the College of Medicine at King Saud University approved an ethical grant (E-21-5986) for this study. All 200 participants signed a written informed consent form before enrollment in this study. This study was conducted in accordance with the principles of the Declaration of Helsinki.

### 2.2. Enrollment of Saudi Participants

Riyadh is the capital city of Saudi Arabia with over 7.5 million residents, and is located in the central region of Saudi Arabia. All enrolled women visited the outpatient clinic of the Department of Obstetrics and Gynecology at King Khalid University Hospital/King Saud University Medical City (KSUMC). In this study, two groups of women were enrolled: (i) women diagnosed with GDM between 24–28 weeks of gestation and (ii) women without any type of diabetes during pregnancy and other diseases (controls/non-GDM). The women in the control group were commonly diagnosed with normal glucose levels. The inclusion criteria for GDM women were based on signing the written informed consent form, elevated glucose levels, and Saudi nationality. In women with GDM, glucose challenge test (GCT) and oral glucose tolerance test (OGTT) were performed. Women who were diagnosed with elevated glucose levels or declined to sign the consent form or with other nationalities were excluded from this study. Participants with normal glucose and BMI levels were considered as controls/non-GDM and were included in this study in comparison with GDM. Women with elevated glucose levels and BMI were excluded from this study as controls. All pregnant women were screened for glucose levels according to the American Diabetes Association criteria [17]. The controls recruited in this study were not on their first pregnancy. We attempted to select women with a mean age suitable for GDM. The enrollment was carried out between November 2021 and October 2022 on the KSUMC premises, and experimental work was performed in the Department of Clinical Laboratory Sciences at KSU (male campus; G-141/1).

### 2.3. Sample Size

In this study, the sample size was calculated for each group based on the following equation, and we decided to enroll a minimum of 100 subjects in each group. Finally, 100 women with GDM and 100 healthy participants were recruited for this study [18].

### 2.4. Diagnosis Screening of GDM and Control Participants

A total of 6 mL of peripheral blood was collected from GDM and control women, and the blood was bifurcated into 4 mL for serum analysis and 2 mL for molecular analysis. Serum analyses included fasting, GCT, OGTT, and lipid profile parameters. Separate serum blood was collected four times during OGTT analysis (F, 1st, 2nd, and 3rd h). In this study, women with GDM were initially screened using the GCT, and women with glucose levels of 7.8 mmol/L or higher were recommended for OGTT test. Based on overnight fasting for a minimum of 8-h and with a recommended diet for at least 72 h, fasting blood was drawn and then 100 g of glucose was given for all the pregnant women and then blood samples were drawn at 1st, 2nd, and 3rd h. If the women were found to exceed the 50% elevated levels of glucose among all OGTT tests, they were considered to have GDM [19]. The control participants did not show elevated glucose levels in either the GCT or OGTT tests (Table 1).

### 2.5. Anthropometric Measurements

All anthropometric parameters were assessed when all participants visited the KSUMC. Anthropometric details, such as age (years), weight (kg), height (cm), and BMI (kg/m^2^) were measured. Hypertension (HTN) was measured using a sphygmomanometer for systolic blood pressure (SBP) and diastolic blood pressure (DBP).

### 2.6. Serum Parameters

Coagulant blood (4 mL) was collected in a fluoride oxalate tube for fasting, GCT (<7.8 mmol/L), and OGTT tests, and the remaining 2 mL of coagulant blood was used for lipid profiling, which was collected in a serum separating tube, which has a special property for activating the clotting factor, clumping the blood cells together. Glucose levels, such as FBG (<5.6 mmol/L), PPBG (<7.8 mmol/L), GCT (<7.8 mmol/L), and OGTT, and lipid profile parameters, such as total cholesterol (TC; normal values = 3.20–5.20 mmol/L), triglycerides (TG; normal range = 0.50–2.27 mmol/L), high-density lipoprotein cholesterol (HDL-C; normal levels = 0.80–1.63 mmol/L), and low-density lipoprotein cholesterol (LDL-C; normal values = 1.81–4.27 mmol/L) were measured.

### 2.7. DNA-PCR Analysis

Two hundred genomic DNA samples were extracted from anticoagulant blood collected in an EDTA tube for molecular analysis using a Qiagen DNA extraction kit, (Qiagen, USA), according to the manufacturer’s protocol. All samples were enumerated on a 1% agarose gel and genomic DNA (*n* = 200) concentration was recalculated using NanoDrop spectrophotometry, converted to 10 ng/µL and stored at −80 °C until molecular analysis was performed. Amplification was performed using polymerase chain reaction (PCR), which was routinely used in the G-141/1 laboratory with a total volume of 50 µL reaction volume as described in our previous publication [20] with a single amendment using 10 ng of genomic DNA. In this study, we selected three genetic variants (rs7903146-*TCF7L2*, rs2237892-*KCNQ1,* and rs5219-*KCNJ11*) associated with β-cell dysfunction in diabetes. SNPs characteristics are listed in Table 2. PCR was carried out in a thermal cycler using 35 cycles a final hold step at 4 °C. Later, to confirm the presence of undigested bands, restriction fragment length polymorphism (RFLP) analysis was performed using the restriction enzymes listed in Table 2.

**Figure 1:**
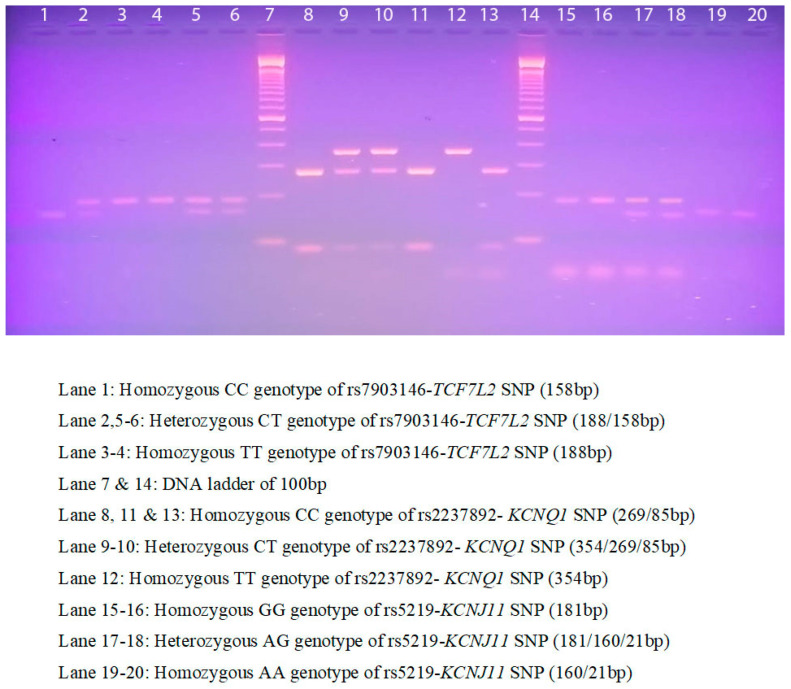
Agarose gel (3%) represents digested products containing the SNPs rs7903146, rs2237892, and rs5219.

RFLP analysis was performed according to previous work [20]. Overnight digestion for 18 h at 37 °C, and both digested and undigested PCR products were run on 2–2.5% ethidium bromide-stained agarose gel electrophoresis between 60–120 min under 100 V, 12 W, and 90 mA conditions. All agarose gels were visualized using a UV transilluminator (Figure 1).

### 2.8. Validation Using Sanger Sequencing

**Figure 2:**
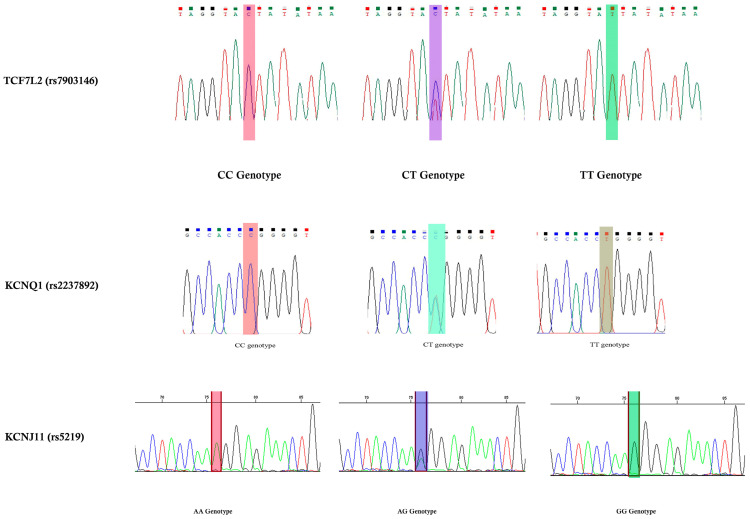
Validation analysis performed for SNPs rs7903146, rs2237892, and rs5219.

In this study, DNA sequencing was performed on 9% of the purified PCR products from both cases and controls (nine GDM and nine controls). To confirm our RFLP results, we performed validation outside of our laboratory. Using dideoxy terminal chemistry and capillary electrophoresis, 182-, 188-, and 354 bp were sequenced for both the forward and reverse reactions. Bidirectional sequencing analysis of the selected PCR products was performed using a big-dye terminator. The sequencing results are described in Figure 2.

### 2.9. Statistical Analysis

The sample size for the GDM and non-GDM groups was calculated using the MedCalc software (Version 20.0, Ostend, Belgium) in a two-group comparison. Numerical variables are described as mean and standard deviation (M ± SD), while categorical variables are described as numbers (*n*) and percentages (%). The clinical data were analyzed with the student’s *z*-test using the SPSS software (version 26.0, Inc., Chicago, IL, USA).

**Figure 3:**
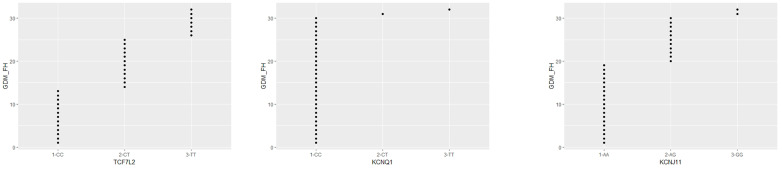
Scatterplot analysis in GDM cases with a family history of GDM.

The Hardy–Weinberg equilibrium (HWE) testing was performed on possible combinations using the chi-square (χ^2^) test. SNPstat software was used to compare genotype and allele frequencies for rs7903146, rs2237892, and rs5219 SNPs between GDM and non-GDM groups for odds ratios (ORs), 95% confidence intervals (CIs), and *p*-values. Multiple logistic regression analysis was used to investigate the potential relationship between the rs7903146, rs2237892, and rs5219 SNPs and covariate frequencies in women with GDM using SPSS software. Using the Jamovi software (Version 2.3.21), a one-way ANOVA analysis with Kruskal–Wallis tests was performed between rs7903146, rs2237892, and rs5219 SNPs and covariate frequencies in women with GDM. Figure 3 shows a scatterplot analysis performed using the R Studio software (version 4.1.2).

## 3. Results

### 3.1. Pre- and Post- Appointment Data for Saudi Women

Anthropometric, biochemical, and clinical details of the 200 Saudi women are shown in. GDM (33.25 ± 6.01) women were documented as older than healthy women (28.17 ± 6.68). Anthropmetric parameters such as age (*p* < 0.0001), weight (*p* = 0.003), and BMI (*p* = 0.0001) were found to be significantly associated with GDM when compared with healthy participants (Table 3).

Hypertensive levels such as SBP (120.33 + 10.54) and DBP (74.09 + 3.22) were elevated in women with GDM (*p* < 0.0001). Biochemical parameters such as FBG (*p* < 0.0001), PPBG (*p* = 0.01), GCT (*p* < 0.0001), OGTT F-3 (*p* < 0.0001), and Hb1Ac levels (*p* < 0.0001) were low in women without GDM and high in women with GDM. Lipid profile parameters were associated with GDM in women with TC (*p* = 0.0001), TG (*p* = 0.0004), and HDLc levels (*p* = 0.0001). In this study, 8% of GDM women were on insulin, and the remaining 92% of women with GDM were recommended a diet. All GDM women with a family history were either from paternal or maternal women in their families (*p* < 0.0001), and 32% of family history was inherited through women pedigree in their families (*p* < 0.0001). Sex (*p* = 1.00), height (*p* = 0.64), and LDLc level (*p* = 0.71) were not associated with this study.

### 3.2. HWE Analysis

The allele frequencies for the rs7903146, rs2237892, and rs5219 SNPs, which were 0.26, 0.12, and 0.09, respectively, are shown in. The rs7903146 SNP was not found to be associated with HWE (χ^2^ = 5.60, *p* = 0.01), whereas the rs2237892 and rs5219 SNPs were (χ^2^ = 2.71; *p* = 0.09, χ^2^ = 2.69, *p* = 0.10). The calculation was performed based on the total number of samples involved in this study (Table 4).

### 3.3. Genotype Frequencies

The genetic association between the rs7903146, rs2237892, and rs5219 SNPs in the *TCF7L2*, *KCNQ1*, and *KCNJ11* genes and the risk of GDM in Saudi women is shown in. In women with GDM, the genotype frequencies for CC, CT, and TT in rs7903146 were 39-, 40-, and 21%, respectively, and 60-, 29-, and 11%, respectively, in women without GDM. The rs223892 SNP predicted CC, CT, and TT in women with GDM at 86-, 10-, and 4%, respectively, and 80-, 17-, and 3% in women without GDM (Table 5).

Finally, the rs5219 SNP showed 62% AA, 32% AG, and 6% GG in GDM cases and 85, 13-, and 2% AA, AG, and GG genotypes, respectively, in healthy participants.

Univariate analysis confirmed a strong statistical significance between rs79013146 (CT vs. CC: OR-2.12 [95%CI: 1.13–3.96]; *p* = 0.01; TT vs. CC: OR-2.93 [95%CI: 1.27–6.75]; *p* = 0.009) and rs5219 (AG vs. AA: OR-3.37 [95%CI: 1.63–6.95]; *p* = 0.0006) SNPs in *TCF7L2* and *KCNJ11* among women with GDM. However, no correlation was found with the rs2237892 SNP in the *KCNQ1* gene (CT vs. CC: OR-0.54 [95%CI: 023–1.26]; *p* = 0.15). The rs7903146 (CT + TT vs. CC: OR-2.34 [95%CI: 1.33–4.13]; *p* = 0.002) and rs5219 (AG + GG vs. AA: OR-3.47 [95%CI: 1.75–6.86]; *p* = 0.0002) SNPs showed a strong association in dominant models; whereas rs2237892 (CT + TT vs. CC: OR-0.65 [95%CI: 0.30–1.37]; *p* = 0.25) showed no association in the GDM group.

### 3.4. Allele Frequencies

Included are separate details for allele frequencies compared between the GDM and healthy groups for the three SNPs (Table 6).

The C allele was found to be 59% and the T allele was 41% in the rs7903146 SNP; whereas nearly one-fourth (25.5%) of the T allele and 74.5% of the C allele were present in the control group. The rs2237892 SNP contained 91% C and 9% T alleles in the GDM group and 88.5% C and 11.5% T alleles in the control group. For the rs5219 SNP, 78% of the A allele and 22% of the G allele were present in the GDM group. The control group included 91.5% A allele and 8.5% G alleles. Statistical analysis confirmed the association of rs7903146 (OR-2.03 [95%CI: 1.32–3.11]; *p* = 0.001) and rs5219 (OR-3.03 [95%CI: 1.66–5.52]; *p* = 0.0001) SNPs; while rs2237892 (OR-0.76 [95%CI: 0.39–1.45]; *p* = 0.40) played no role in allele frequencies between GDM and non-GDM women.

### 3.5. Multiple Logistic Regression Analysis

Multiple logistic regression analysis was performed between the clinicopathological features of GDM subjects and the three SNPs involved in this study. Logistic regression analysis confirmed that only the HDLc parameter showed a positive association (*p* = 0.04) with the combination of three SNPs. The F and T values were found to be 2.75 and 14.27, respectively. However, the other covariates showed no association, as shown in Table 7.

### 3.6. ANOVA Analysis

In this study, the results of the relationship between the SNPs (rs7903146, rs2237892, and rs5219) and the 17 covariates listed in were studied using ANOVA analysis and differentiated with three different genotypes in the SNPs, as summarized in Table 8.

Age (33.92 ± 6.37), SBP (121.74 ± 10.91), FBG (6.10 ± 1.41), PPBG (12.08 ± 2.32), GCT (9.28 ± 1.12), OGTT-F (6.74 ± 2.38), OGTT-2 (9.69 ± 1.77), and lipid profile parameters: TC (5.96 ± 1.49), TG (2.71 ± 2.74), HDLc (1.04 ± 0.46), and LDLc (3.87 ± 1.13) levels are higher in CC genotypes of the rs7903146 SNP. The CT genotype had high DBP (74.82 ± 3.48) and Hb1Ac (5.47 ± 0.28) levels. Weight (83.37 ± 10.97), BMI (33.34 ± 4.27), OGTT-1 (11.02 ± 1.42), and OGTT-3 (6.29 ± 1.81) levels were all elevated in the TT genotype. ANOVA confirmed that weight, BMI, and PPBG were associated with the rs7903146 SNP in GDM patients. In the rs2237892 SNP FBG (5.99 ± 1.27), PPBG (9.70 ± 17.78), GCT (9.36 ± 1.08), and Hb1Ac (5.43 ± 0.35) levels were found to be high in the CC genotype. Age (35.90 ± 5.74), weight (84.99 ± 11.84), BMI (35.20 ± 4.10), SBP (121.80 ± 12.17), OGTT-F (6.97 ± 2.56), TC (6.61 ± 1.94), TG (3.24 ± 1.61), and HDLc (1.14 ± 0.53) levels are higher in the heterozygous variant. The TT genotype had increased DBP (75.00 ± 3.92), OGTT: 1–3 (11.08 ± 0.73, 10.55 ± 0.29 & 6.23 ± 2.74), and LDLc (4.21 ± 0.47) levels in GDM patients with the rs2237892 SNP. ANOVA confirmed that rs2237892 was strongly associated with BMI (*p* = 0.03). In the final SNP rs5219, Age (33.69 ± 6.20), BMI (32.23 ± 4.64), SBP (120.92 ± 10.98), DBP (74.32 ± 3.42), GCT (9.38 ± 1.02), OGTT: F (6.46 ± 1.97), OGTT: 1–2 (10.73 ± 1.92 & 9.34 ± 1.55), Hb1Ac (5.44 ± 0.36), TG (2.61 ± 2.47), and LDLc (3.87 ± 1.04) levels were found to be elevated in the AA genotype. The AG genotype had higher levels of PPBG (12.95 ± 2.06), OGTT: 2 (9.34 ± 1.88), and TC (5.98 ± 1.44). Weight (82.73 ± 10.89), FBG (6.17 ± 1.43), OGTT: 3 (6.08 ± 1.43), and HDLc (1.22 ± 0.43) levels were all high in the GG genotype. In any of the covariates in the rs5219 SNP, there was no association found using the ANOVA analysis (*p* > 0.05).

## 4. Discussion

GDM can also occur because of hyperglycemia, which has an antagonistic effect on insulin and leads to insulin resistance. Pancreatic β-cells play an important role in maintaining virtually constant compensation for insulin resistance at a lower level than that in normal pregnant women because insulin levels in pregnant women with GDM can be low or high [21]. Understanding the in vivo correlation between candidate genes and complicated diseases may be possible through minimal and large-sample unbiased epidemiological investigations of propensity gene polymorphisms. This study aimed to investigate the role of rs7903146, rs2238792, and rs5219 SNPs in Saudi women with GDM during pregnancy. The genotype and allele frequencies of these results confirm that rs7903146 and rs5219 SNPs have 2–3-fold higher risks in women with GDM. Additionally, ANOVA analysis confirmed that weight (*p* = 0.02), BMI (*p* = 0.01), and PPBG (*p* = 0.003) were associated with rs7903146 and BMI (*p* = 0.03) was associated with rs2237892 SNPs in GDM subjects. HWE analysis was not associated with the rs7903146 SNP in this study, but it was strongly associated with genotype, allele frequencies, logistic regression, and ANOVA analysis (*p* < 0.05). A family history of GDM was found in 40.6% of CC genotypes, 37.5% of CT genotypes, and 21.9% of TT genotypes in the rs7903146. Among the rs2237892 SNP, CC genotypes were present in 93.8% of GDM cases, whereas the CT and TT genotypes accounted for 1.3% of GDM cases. Finally, those with the GG genotype had a 6.2% higher risk of developing GDM than those with the AA and AG genotypes (59.4% AA and 34.4% AG, respectively). Normal genotypes were 64.6%, heterozygous 25%, and homozygous variant genotypes at 10.4% (Table 9). The family history of GDM women is shown via scatterplot analysis in Figure 3.

The pathological characteristics of GDM and T2DM are similar; however, the stages of disease development vary. One of the dissimilitudes of different types of diabetes is that GDM develops during pregnancy and resolves after delivery, whereas T2DM is a chronological disorder.

Among numerous genetic loci, *TCF7L2* (rs7903146), *KCNQ1* (rs2237892), and *KCNJ11* (rs5219) are associated with β-cell function, insulin resistance, and insulin action in GDM [22]. *TCF7L2* was found to increase the risk of GDM and was confirmed to be a reliable predictor of T2DM. *TCF7L2* is required for the Wnt signaling pathway to function. In addition to playing a significant role in β-cell development and function, it is also a key regulator of glucose homeostasis [23]. *KCNQ1* is involved in pancreas and kidney function, and genetic variants are intimately associated with T2DM, insulin secretion, and impaired fasting blood glucose levels. K_ATP_ channels in the *KCNJ11* gene are abundant in the pancreas and play an important role in regulating insulin secretion in pancreatic β-cells. *TCF7L2*, *KCNQ1*, and *KCNJ11* are associated with the onset of T2DM and vascular complications [15]. However, these three genes are also associated with GDM. Earlier studies have shown that GDM and T2DM have common genetic variants, with comparable impact sizes on the same risk alleles [24]. This statement, however, was consistent with those of previous studies in the Asian Indian population. Khan et al. conducted their research on three different types of diabetes, including T2DM, GDM, and post-transplant diabetes mellitus (PTDM), using rs7903146, rs228228, and rs5210. SNPs in the *TCF7L2*, *KCNQ1*, and *KCNJ11* genes were studied, and the results confirmed that these three SNPs were associated with all types of diabetes studied, including T2DM, GDM, and PTDM. However, in ethe present study, the rs7903146 SNP was commonly studied and found to be associated, whereas rs228228 and rs5210 were found to be associated with the *KCNQ1* and *KCNJ11* genes in a study by Khan et al., in which we chose different SNPs (rs2238792 and rs5219) in Saudi women with GDM.

Replication of case-control studies was carried out in different populations of women with GDM among the rs7903146 [25,26,27,28,29,30], rs2237892 [31,32,33,34,35], and rs5219 [35,36,37,38] SNPs. Meta-analysis studies have been carried out with rs7903146 [39,40,41,42,43], rs2237892 [42,44] and rs5219 SNPs [42] in women with GDM. This study found that the minor allele frequency (MAF) of rs7903146 was 41%, whereas other studies have shown inconsistent results across countries and regions. For example, the frequency of this allele is 10.5% in Malaysia [45], 3.3% in South Korea [46], 2% in China [29], 36.4% in Qatar [47], 33% in Poland [25], 36.2% in Spain [48], 74% in Lithuania [49], 38.8% in India, 24% in Bangladesh [50], 23% in Mexico [27], 48.4% in Australia [51], and 27.6% in Denmark [52]. The MAF of the rs2237892 SNP in this population was determined to be 9%, with a prevalence of 8.3% in Poland [35], which is quite close to our study. The populations of other countries were documented as 34% [32] and 34.2% [53] in Korea, 36% in Japan [54], and 28.9% [44] and 27.8% [55] in China. The rs5219 SNP was found in our study at a rate of 22%, which is comparable to that in the Greek population of 20.9% [56]. The prevalence was 26% [38] and 38% [57] in the Indian population, 36.5% in the Polish population [35], 59.3% in the Russian population [36], 40% in the Danish population [52], and 42.2% in the Swedish population [58]. However, rs7903146 SNP was not associated in T2DM subjects from Eastern provenience region in Saudi Arabia. However, rs12255372 and rs4506565 SNPs was associated [59]. Ding et al. [60] in his meta-analysis confirmed a strong association in the rs7903146 SNP in T2DM patients of Caucasian, East/South Asian, and other ethnicities in global studies. A case-control study in Egyptian women in rs703146 and rs12255372 SNPs in TCF7L2 gene in GDM women revealed a positive association in both SNPs [26].

In this study, 8% of the women with GDM were on medication, while the remaining 92% were on a diet. All 8% of women with GDM had a 2% family history of the rs7903146 SNP heterozygous variant (CT) and homozygous normal genotypes in the rs2237892 (CC) and rs5219 (AA) SNPs. GDM women were found to be obese in 65% (mean age, 34.25 ± 3.15), overweight in 27% (mean age, 28.11 ± 1.37), and the remaining 8% (mean age, 22.91 ± 1.83). Meta-analysis studies of rs7903146 [61,62] and rs2237892 [63] SNPs in obesity showed a strong association. The ANOVA analysis of this study results confirmed that both rs79033146 and rs2237892 SNPs were associated with BMI levels. HTN levels were found to be normal in all genotype groups, and no significant difference was found in this study. Notably, no meta-analysis has documented the association between the three SNPs studied in this study. FBG, GCT, OGTT-F:3h, and Hb1Ac levels were not associated with the three SNPs studied; however, PPBG levels were associated with rs7903146 SNP (*p* = 0.003).

This study has several limitations and strengths. One of the limitations of this study was the small sample size due to which rs7903146 SNP was not accordance with HWE analysis and the inclusion of only a single SNP from each gene. Another limitation of this study was the lack of measurement of serum levels. The other limitation of this study was missing of documenting the evidence of pregnancy weight gain. The final limitations of this study include the absence of parameters related to pregnancy monitoring and outcome, delivery complications, fetal status, and neonatal outcome. The strength of this study is that it included all Saudi women who had been diagnosed with GDM, and it has expanded the maximum statistics applicable to this study.

## 5. Conclusions

Our study of Saudi women showed strong genotype and allelic associations with rs7903146 and rs5219 SNPs in GDM. In addition, this study confirms the susceptibility of Saudi women to GDM. According to the statistical findings of this study, these three SNPs may be involved in the development of GDM. Further global studies should be conducted to validate the findings of the current study. However, rs7903146 SNP was not associated in Saudi T2DM patients from Eastern provenience region of Saudi Arabia. A similar association was found in three SNPs in the global population in GDM women, including Caucasian, Asian, and other ethnicities.

## Figures and Tables

**Table 1:** OGTT values for diagnosis of GDM women.

Threshold for Diagnosis	Molarity Values (mmol/L)	Concentrated Values (mg/dL)
Fasting	5.3 mmol/L	95 mg/dL
First Hour	10.0 mmol/L	180 mg/dL
Second Hour	8.6 mmol/L	155 mg/dL
Third Hour	7.8 mmol/L	140 mg/dL

**Table 2:** List of SNPs and related details involved in this study.

Gene	rs Number	SNP	Forward Primer	Reverse Primer	PCR Size	Annealing Temperature	Restriction Enzyme	Digested Products
*TCF7L2*	rs7903146	C-T	ACAATTAGAGAGCTAAGCACTTTTTAGGTA	GTGAAGTGCCCAAGCTTCTC	188 bp	60 °C	RsaI	C-159/29 bp T-188 bp
*KCNQ1*	rs2237892	C-T	CTTGTGCCCTTGTCACCCAC	GGCTTCCAGCCTCCAAGCGT	354 bp	64 °C	HpaII	C-269/85 bp T-354 bp
*KCNJ11*	rs5219	A-G	GTGCCAACCGAGAGGACTCTGCA	TGGCGGGCACGGTACCTAAGCT	181 bp	62 °C	BfaI	A-160/21 bp G-181 bp

**Table 3:** Clinicodemographic data obtained between GDM and control participants.

	Controls (*n* = 100)	GDM (*n* = 100)	*p* Value
Age	28.17 ± 6.68	33.25 ± 6.01	<0.0001
Gender	0/100	0/100	1.00
Weight	72.81 ± 11.79	79.13 ± 12.84	0.003
Height	157.87 ± 4.98	158.21 ± 5.45	0.64
BMI	29.27 ± 4.18	31.68 ± 4.60	0.0001
SBP	109.41 ± 11.91	120.33 ± 10.54	<0.0001
DBP	63.48 ± 8.72	74.09 ± 3.22	<0.0001
FBG	4.28 ± 0.42	5.91 ± 1.22	<0.0001
PPBG	4.56 ± 11.13	9.44 ± 16.51	0.01
GCT	6.25 ± 1.06	9.28 ± 1.07	<0.0001
OGTT (F)	4.84 ± 0.61	6.61 ± 2.24	<0.0001
OGTT (1)	7.17 ± 1.74	10.59 ± 1.84	<0.0001
OGTT (2)	6.21 ± 1.56	9.33 ± 1.62	<0.0001
OGTT (3)	4.21 ± 1.16	5.89 ± 1.53	<0.0001
Hb1Ac	4.81 ± 0.32	5.42 ± 0.35	<0.0001
TC	5.11 ± 1.15	5.78 ± 1.32	0.0001
TG	1.62 ± 0.92	2.42 ± 2.03	0.0004
HDL-C	0.68 ± 0.25	0.94 ± 0.41	0.0001
LDL-C	3.74 ± 0.98	3.79 ± 0.96	0.71
Medication (Insulin)	0	08 (08%)	NA
Family History of T2DM	26 (26%)	100 (100%)	<0.0001
Family History of GDM	10 (10%)	32 (32%)	<0.0001

**Table 4:** HWE analysis with the three SNPs in observed genotypes.

SNPs	Minor Allele	Allele Frequency	X^2^	HW *p*-Value
rs7903146	T	0.26	5.60	0.01
rs2237892	T	0.12	2.71	0.09
rs5219	G	0.09	2.69	0.10

With HWE, *p* < 0.05 is considered non-significant.

**Table 5:** Genotyping analysis of three SNPs studied in GDM and non-GDM women.

Gene (rs Number)	Genotypes	GDM (*n* = 100)	Non-GDM (*n* = 100)	OR (95%CI) and *p* Value
*TCF7L2* (rs7903146)	CC	39 (39%)	60 (60%)	Reference
	CT	40 (40%)	29 (29%)	2.12 (1.13–3.96) and *p* = 0.01
	TT	21 (21%)	11 (11%)	2.93 (1.27–6.75) and *p* = 0.009
	CT + TT vs. CC	61 (61%)	40 (40%)	2.34 (1.33–4.13) and *p* = 0.002
	TT+CC vs. CT	60 (60%)	71 (71%)	0.61 (0.34–1.10) and *p* = 0.10
	CC+CT vs. TT	79 (79%)	89 (89%)	0.46 (0.21–1.02) and *p* = 0.05
*KCNQ1* (rs2237892)	CC	86 (86%)	80 (80%)	Reference
	CT	10 (10%)	17 (17%)	0.54 (0.23–1.26) and *p* = 0.15
	TT	04 (04%)	03 (03%)	1.24 (0.26–5.71) and *p* = 0.78
	CT + TT vs. CC	14 (14%)	20 (20%)	0.65 (0.30–1.37) and *p* = 0.25
	TT+CC vs. CT	90 (90%)	83 (83%)	1.84 (0.79–4.25) and *p* = 0.14
	CC+CT vs. TT	96 (96%)	97 (97%)	0.74 (0.16–3.41) and *p* = 0.70
*KCNJ11* (rs5219)	AA	62 (62%)	85 (85%)	Reference
	AG	32 (32%)	13 (13%)	3.37 (1.63–6.95) and *p* = 0.0006
	GG	06 (06%)	02 (02%)	4.11 (0.81–21.06) and *p* = 0.06
	AG+GG vs. AA	38 (38%)	15 (15%)	3.47 (1.75–6.86) and *p* = 0.0002
	GG+AA vs. AG	68 (68%)	87 (87%)	0.31 (0.15–0.65) and *p* = 0.001
	AA+AG vs. GG	94 (94%)	98 (98%)	0.31 (0.06–1.62) and *p* = 0.14

**Table 6:** Allele frequencies between GDM and non-GDM subjects in 3 SNPs.

Gene (rs Number)	Genotypes	GDM (*n* = 100)	Control (*n* = 100)	OR (95%CI) and *p* Value
*TCF7L2* (rs7903146)	C	118 (0.59%)	149 (74.5%)	Reference
	T	82 (0.41%)	51 (25.5%)	2.03 (1.32–3.11) and *p* = 0.001
*KCNQ1* (rs2237892)	C	182 (0.91%)	177 (88.5%)	Reference
	T	18 (0.09%)	23 (11.5%)	0.76 (0.39–1.45) and *p* = 0.40
*KCNJ11* (rs5219)	A	156 (0.78%)	183 (91.5%)	Reference
	G	44 (0.22%)	17 (8.5%)	3.03 (1.66–5.52) and *p* = 0.0001

**Table 7:** Multiple logistic regression analysis.

Covariates	R-Value ^a^	Adjusted R Value	Unstandardized β-Coefficient for rs7903146	Unstandardized β-Coefficient for rs2237892	Unstandardized β-Coefficient for rs5219	F	*t*-Value	*p* Value ^b^
Age	0.241	0.029	−0.818	2.358	−1.500	1.978	35.361	0.122
Weight	0.171	0.029	−0.376	3.766	−2.268	0.964	38.068	0.413
BMI	0.209	0.014	−0.139	1.733	−0.893	1.457	42.788	0.231
SBP	0.127	0.016	−1.427	1.429	−1.182	0.527	70.356	0.665
DBP	0.167	−0.003	0.503	0.235	−0.578	0.917	140.51	0.436
FBG	0.145	−0.009	−0.108	−0.286	0.057	0.690	29.950	0.561
PPBG	0.158	−0.005	−2.398	−0.373	3.140	0.823	3.744	0.484
GCT	0.204	0.011	0.024	−0.403	−0.168	1.371	53.857	0.256
OGTT (F)	0.072	−0.026	−0.124	0.161	0.207	0.169	17.759	0.917
OGTT (1)	0.125	−0.015	−0.092	0.312	−0.296	0.508	35.393	0.678
OGTT (2)	0.193	0.007	−0.339	0.540	−0.003	1.238	36.011	−0.152
OGTT (3)	0.148	−0.009	0.303	−0.094	0.060	0.716	22.491	0.545
Hb1Ac	0.117	−0.017	−0.010	−0.074	−0.024	0.444	94.096	0.722
TC	0.203	0.011	−0.334	0.352	0.086	1.378	27.721	0.254
TG	0.216	0.017	−0.454	0.368	−0.438	1.570	8.894	0.202
HDL-C	0.282	0.051	−0.999	0.039	0.156	2.755	14.277	0.047
LDL-C	0.146	−0.009	−0.080	−0.088	−0.181	0.698	25.059	0.556

^a^ Predictors: (Constants), TCF7L2 -rs7903146, KCNQ1-rs2237892 and KCNJ11-rs5219. ^b^ Dependent variables are listed in covariates.

**Table 8:** ANOVA analysis variance between SNPs and clinical/biochemical characteristics.

	*TCF7L2* (rs7903146)				*KCNQ1* (rs2237892)				*KCNJ11* (rs5219)			
	CC (*n* = 39)	CT (*n* = 40)	TT (*n* = 21)	*p*	CC (*n* = 86)	CT (*n* = 10)	TT (*n* = 04)	*p*	AA (*n* = 62)	AG (*n* = 32)	GG (*n* = 06)	*p*
Age	33.92 ± 6.37	32.62 ± 6.07	33.19 ± 5.33	0.63	32.84 ± 6.06	35.90 ± 5.74	35.50 ± 4.20	0.23	33.69 ± 6.20	33.22 ± 5.78	28.83 ± 3.87	0.16
Weight	81.18 ± 13.55	74.90 ± 12.09	83.37 ± 10.97	0.02	78.33 ± 12.86	84.99 ± 11.84	81.79 ± 14.13	0.27	80.77 ± 13.03	75.29 ± 12.28	82.73 ± 10.89	0.11
BMI	32.41 ± 4.37	30.11 ± 4.62	33.34 ± 4.27	0.01	31.27 ± 4.55	35.20 ± 4.10	31.95 ± 3.87	0.03	32.23 ± 4.64	30.56 ± 4.61	32.12 ± 3.67	0.24
SBP	121.74 ± 10.91	119.32 ± 9.22	119.62 ± 12.32	0.56	120.14 ± 10.21	121.80 ± 12.17	120.75 ± 16.26	0.89	120.92 ± 10.98	119.44 ± 9.69	119.00 ± 11.73	0.77
DBP	73.33 ± 2.65	74.82 ± 3.48	74.10 ± 3.51	0.12	74.02 ± 3.33	74.30 ± 1.95	75.00 ± 3.92	0.82	74.32 ± 3.42	73.81 ± 2.93	73.17 ± 2.79	0.59
FBG	6.10 ± 1.41	5.75 ± 1.09	5.86 ± 1.12	0.44	5.99 ± 1.27	5.42 ± 0.83	5.60 ± 0.93	0.33	5.91 ± 1.38	5.87 ± 0.87	6.17 ± 1.43	0.86
PPBG	12.08 ± 2.32	7.62 ± 1.65	8.00 ± 1.87	0.003	9.70 ± 17.78	7.75 ± 1.30	8.15 ± 2.32	0.92	7.65 ± 1.68	12.95 ± 29.06	9.25 ± 1.38	0.33
GCT	9.28 ± 1.12	9.34 ± 1.08	9.17 ± 1.02	0.84	9.36 ± 1.08	8.86 ± 0.96	8.68 ± 1.01	0.19	9.38 ± 1.02	9.13 ± 0.97	9.15 ± 2.04	0.54
OGTT (F)	6.74 ± 2.38	6.47 ± 2.16	6.62 ± 2.27	0.86	6.57 ± 2.20	6.97 ± 2.56	6.50 ± 3.11	0.86	6.46 ± 1.97	6.96 ± 2.73	6.30 ± 2.13	0.56
OGTT (1)	10.89 ± 2.07	10.07 ± 1.73	11.02 ± 1.42	0.06	10.55 ± 1.89	10.80 ± 1.83	11.08 ± 0.73	0.80	10.73 ± 1.92	10.41 ± 1.85	10.20 ± 0.86	0.63
OGTT (2)	9.69 ± 1.77	8.99 ± 1.56	9.32 ± 1.39	0.16	9.29 ± 1.66	9.28 ± 1.55	10.55 ± 0.29	0.31	9.34 ± 1.55	9.34 ± 1.88	9.32 ± 1.06	0.99
OGTT (3)	5.68 ± 1.52	5.89 ± 1.38	6.29 ± 1.81	0.34	5.90 ± 1.46	5.71 ± 1.75	6.23 ± 2.74	0.84	5.87 ± 1.46	5.91 ± 1.72	6.08 ± 1.43	0.94
Hb1Ac	5.41 ± 0.42	5.47 ± 0.28	5.34 ± 0.33	0.38	5.43 ± 0.35	5.42 ± 0.41	5.20 ± 0.27	0.44	5.44 ± 0.36	5.39 ± 0.31	5.42 ± 0.54	0.96
TC	5.96 ± 1.49	5.82 ± 1.18	5.38 ± 1.24	0.26	5.70 ± 1.18	6.61 ± 1.94	5.37 ± 2.21	0.10	5.27 ± 1.27	5.98 ± 1.44	5.49 ± 1.31	0.05
TG	2.71 ± 2.74	2.43 ± 1.54	1.85 ± 1.02	0.29	2.35 ± 2.11	3.24 ± 1.61	1.87 ± 0.46	0.36	2.61 ± 2.47	2.20 ± 0.89	1.64 ± 0.86	0.40
HDL-C	1.04 ± 0.46	0.85 ± 0.35	0.90 ± 0.43	0.11	0.93 ± 0.40	1.14 ± 0.53	0.72 ± 0.45	0.18	0.88 ± 0.38	1.01 ± 0.46	1.22 ± 0.43	0.08
LDL-C	3.87 ± 1.13	3.77 ± 0.83	3.68 ± 0.89	0.75	3.85 ± 0.97	3.17 ± 0.84	4.21 ± 0.47	0.07	3.87 ± 1.04	3.70 ± 0.86	3.46 ± 0.67	0.50

**Table 9:** Representation of family history in the form of different genotypes in SNPs.

Genes/SNPs	Normal Genotypes (CC/AA)	Heterozygous (CT/AG)	Homozygous Variant (TT/GG)
*TCF7L2* (rs7903146)	13 (40.6%)	12 (37.5%)	07 (21.9%)
*KCNQ1* (rs2237892)	30 (93.8%)	01 (3.1%)	01 (3.1%)
*KCNJ11* (rs5219)	19 (59.4%)	11 (34.4%)	02 (6.2%)
Total Genotypes	62 (64.6%)	24 (25%)	10 (10.4%)

## Data Availability

Not applicable.
